# Geniposidic Acid Reduces Lipid Accumulation and Is Associated with Altered AMPK Phosphorylation in 3T3-L1 Adipocytes

**DOI:** 10.3390/molecules31213806

**Published:** 2026-10-22

**Authors:** Se-Yeong Park, Chanhyeok Lee, Hyocheol Bae

**Affiliations:** Department of Convergent Biotechnology and Advanced Materials Science, College of Life Sciences, Kyung Hee University, Yongin 17104, Republic of Korea; hhjshh12@khu.ac.kr (S.-Y.P.); iede1998@khu.ac.kr (C.L.)

**Keywords:** geniposidic acid, obesity, adipogenesis, lipid accumulation, AMPK, 3T3-L1 cells

## Abstract

Obesity is a chronic metabolic disorder characterized by excessive adipose tissue accumulation and dysregulated lipid metabolism. This study investigated the effects of geniposidic acid (GA) on intracellular lipid accumulation and associated molecular responses during 3T3-L1 adipocyte differentiation. MDI-induced cells were treated with GA (2.5–10 μg/mL) and evaluated using lipid staining, RT-qPCR, Western blot, and JC-1 assays, alongside network pharmacology and computational analyses. Network analysis identified 57 overlapping GA-associated and obesity-related candidate targets, including associations with PPAR signaling. GA reduced intracellular lipid accumulation, with significant reductions at 5 and 10 μg/mL. Gene and protein expression analyses revealed decreases in adipogenic and lipogenic markers and increases in selected fatty acid metabolism-related markers. GA also attenuated the loss of mitochondrial membrane potential at 10 μg/mL. The p-AMPKα/AMPKα ratio was 1.28-fold that of the MDI-treated group at 10 μg/mL, whereas lower concentrations did not show a consistent increase. Docking scores for PPARα and PPARγ were −7.634 and −6.735 kcal/mol, respectively, and the modeled complexes were evaluated over 200 ns. These findings suggest that GA may reduce lipid accumulation and alter metabolic markers, supporting its potential as a natural bioactive compound for further investigation in obesity-related metabolic dysfunction.

## 1. Introduction

Obesity is a chronic, highly recurrent, multifactorial disease characterized by excessive accumulation of adipose tissue and resulting from complex interactions among dietary habits, genetic factors, socioeconomic environments, and other factors; it is a major public health issue that is rapidly increasing worldwide [1,2,3]. According to a study analyzing obesity prevalence in 200 countries from 1990 to 2022, the global adult obesity rate has more than doubled, with more than 1 billion people living with obesity or overweight worldwide [3,4]. The primary recommended treatment for obesity is lifestyle modification based on dietary control and exercise, supplemented by non-surgical drug therapy and, in limited cases, surgical procedures [5,6].

Orlistat and GLP-1 receptor agonists are widely used and have demonstrated clinically significant effects on weight loss. Orlistat reduces dietary fat absorption by inhibiting gastrointestinal lipases, resulting in increased excretion of unabsorbed fat in the stool, while GLP-1 receptor agonists work through mechanisms such as appetite suppression, delayed gastric emptying, enhanced insulin secretion, and improved blood glucose control [7,8,9,10]. However, gastrointestinal side effects, high treatment costs, the need for long-term use, the requirement to take additional vitamin supplements, and weight regain after discontinuing treatment remain significant limitations of current treatment methods [11,12,13]. Consequently, there is significant interest in the development of natural-product-based anti-obesity compounds that can regulate adipogenesis and lipid metabolism while potentially causing fewer side effects.

Adipose tissue expansion is primarily driven by adipocyte hypertrophy and proliferation, which are closely associated with adipocyte differentiation and lipid accumulation [14]. AMP-activated protein kinase (AMPK), a key regulator of cellular energy homeostasis, suppresses lipid synthesis and promotes fatty acid oxidation by regulating downstream metabolic targets [15]. Adipocyte differentiation is generally regulated by several adipogenic transcription factors, including CCAAT/enhancer-binding protein β (*Cebpb*), CCAAT/enhancer-binding protein α (*Cebpa*), and peroxisome proliferator-activated receptor γ (*Pparg*). During adipogenesis, pre-adipocytes undergo a differentiation process; following the induction of *CEBPβ*, increased expression of *CEBPα* and *PPARγ* promotes adipocyte differentiation and lipid accumulation [16]. In addition, lipogenic factors such as sterol regulatory element-binding protein-1c (*Srebp1c*), fatty acid synthase (*Fasn*), and acetyl-CoA carboxylase 1 (*Acaca*) contribute to lipid storage by promoting fatty acid synthesis and triglyceride (TG) accumulation [15,17]. Therefore, the regulation of adipogenic transcription factors and AMPK-related signaling pathways is gaining attention as a key preventive and therapeutic target for obesity management.

**Figure 1:**
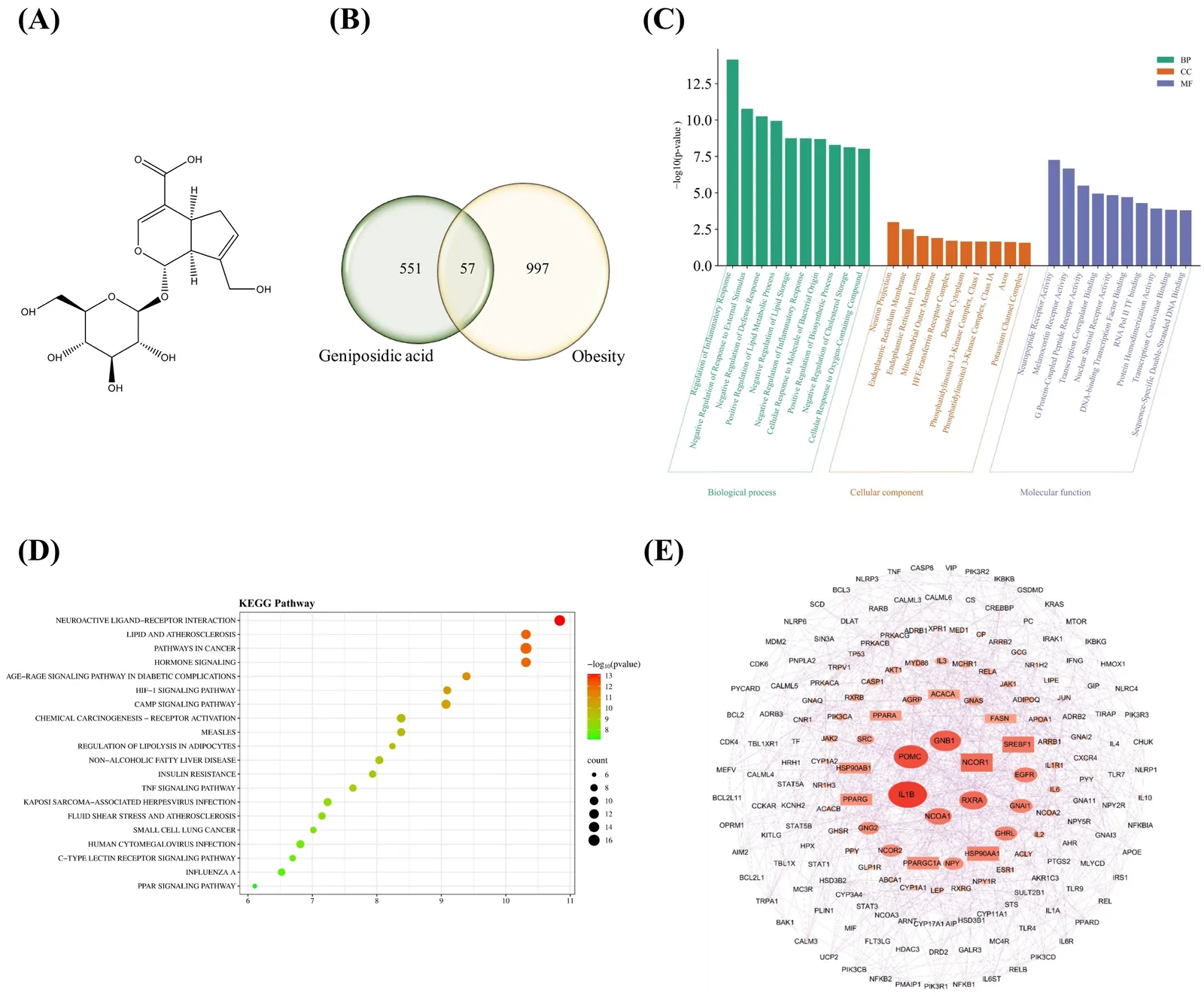
Network pharmacology analysis of obesity-related targets and signaling pathways associated with GA. (**A**) Chemical structure of GA. (**B**) Identification of 57 common targets shared between GA and obesity-associated genes through intersection analysis. (**C**) GO enrichment analysis of the overlapping targets in Biological Process (BP), Cellular Component (CC), and Molecular Function (MF) categories. The top 10 terms in each category were selected based on nominal *p*-values and are presented as −log_10_(*p*-value). Cellular Component terms did not remain statistically significant after multiple-testing correction (adjusted *p* ≥ 0.05). (**D**) KEGG pathway enrichment analysis showing obesity-related pathways enriched among the common targets. Statistical significance was evaluated using adjusted *p*-values, with adjusted *p* < 0.05 considered statistically significant. (**E**) Subnetwork extracted from the expanded PPI network based on degree values greater than the network average (degree ≥ 10). The displayed network contains 179 nodes and 882 edges. Node size and color intensity are proportional to degree values, highlighting highly connected proteins within the network.

Iridoid glycosides, which are widely found in medicinal plants, are known to exhibit various biological activities, including the reduction in oxidative stress, the regulation of inflammatory responses, and the modulation of lipid metabolism [18,19]. Among these, geniposidic acid (GA) is an iridoid glycoside containing a glucose moiety attached to a cyclopentapyran skeleton (Figure 1A) has been identified as a major constituent of *Plantago asiatica* L. [20]. Previous studies have reported that GA-containing *P. asiatica* seed extracts improved lipid and glucose metabolism in high-fat-diet-induced obese mice [20,21]. More recently, Tang et al. demonstrated that purified GA reduced serum lipid levels and hepatic lipid accumulation in a high-fat-diet-induced hyperlipidemia mouse model [22]. Regarding pharmacokinetics, GA has been detected in rat plasma following oral administration of herbal extracts, with systemic exposure varying according to extract processing methods [23]. Nevertheless, the available extract-based pharmacokinetic data do not establish the absolute oral bioavailability of purified GA or its systemic exposure in humans. Therefore, although previous findings suggest a potential role for GA in lipid metabolic regulation, its direct effects on adipocyte differentiation and the associated molecular responses remain insufficiently characterized.

Therefore, the present study investigated the potential effects of GA on lipid accumulation and adipocyte differentiation using MDI-induced 3T3-L1 adipocytes. Network pharmacology analysis was performed to explore potential associations between GA-related targets and obesity-associated signaling pathways and to generate hypotheses regarding their biological relevance. In particular, the identification of PPAR signaling and related metabolic factors suggested that GA treatment might be associated with alterations in PPAR-related factors and the expression of genes involved in adipogenesis and lipid metabolism. To examine this possibility, intracellular lipid accumulation and the expression of selected adipogenic and lipid metabolism-related genes and proteins were evaluated. Furthermore, molecular docking and molecular dynamics simulations were conducted to explore potential interactions between GA and PPARα and PPARγ. Collectively, these findings provide insights into the cellular responses associated with GA treatment and suggest its potential relevance to adipocyte differentiation and lipid metabolism, warranting further investigation in the context of obesity-related metabolic dysfunction.

## 2. Results

### 2.1. Network Pharmacology Identified Obesity-Related Targets and Signaling Pathways Associated with GA

To explore potential molecular associations between GA and obesity-related signaling pathways, a network pharmacology-based analysis was performed. A total of 608 potential targets of GA were collected from the GeneCards, STITCH, SwissTargetPrediction, and TargetNet databases, whereas 1054 obesity-associated genes were obtained from the GeneCards and DisGeNET databases. Intersection analysis identified 57 common targets shared between GA and obesity-related genes (Figure 1B), suggesting potential associations between GA and obesity-related biological processes. GO enrichment analysis was performed to investigate the functional characteristics of the overlapping targets (Figure 1C). Within the Biological Process category, several terms associated with inflammatory regulation and lipid metabolism showed statistically significant enrichment after multiple-testing correction (adjusted *p* < 0.05), including regulation of inflammatory response, positive regulation of lipid metabolic process, and negative regulation of defense response. Molecular Function analysis also identified significantly enriched terms associated with receptor activity and transcriptional regulation, including neuropeptide receptor activity, melanocortin receptor activity, G protein-coupled peptide receptor activity, and transcription coregulator binding. In contrast, although several Cellular Component terms, including neuron projection and endoplasmic reticulum membrane, exhibited nominal enrichment, these associations did not remain statistically significant after multiple-testing correction (adjusted *p* ≥ 0.05). Collectively, these findings suggested potential associations between the overlapping targets and biological processes related to inflammation and lipid metabolism, together with receptor-mediated signaling and transcriptional regulation. KEGG pathway enrichment analysis further demonstrated that the overlapping targets were significantly associated with multiple obesity-related signaling pathways (Figure 1D). Among the enriched pathways, the PPAR signaling pathway, cAMP signaling pathway, regulation of lipolysis in adipocytes, insulin resistance, lipid and atherosclerosis, and non-alcoholic fatty liver disease (NAFLD) were identified. Notably, the cAMP signaling pathway exhibited a relatively high enrichment significance, while pathways involved in adipocyte lipid metabolism and energy regulation were also prominently represented. To investigate the interactions among the overlapping targets, an expanded PPI network was constructed by integrating the 57 common targets with their functionally associated protein partners obtained from the STRING database. The resulting network consisted of 454 nodes and 2195 edges (Figure S1). Based on network topology analysis, proteins with degree values greater than the network average (degree ≥ 10) were selected, yielding a subnetwork containing 179 nodes and 882 edges (Figure 1E). Several proteins, including *IL1B*, *POMC*, *GNB1*, *NCOR1*, *RXRA*, *EGFR*, *PPARG*, and *PPARA*, occupied central positions within the network. Furthermore, proteins closely associated with adipogenesis and lipid metabolism, such as *SREBF1*, *PPARGC1A*, *ACACA*, and *FASN*, were also identified within highly interconnected regions. *PPARG* and *PPARA* exhibited extensive interactions with proteins involved in adipogenesis, lipid metabolism, inflammatory responses, and energy homeostasis. These observations suggested a potential association between GA-related candidate targets and *PPAR*-associated metabolic pathways. Based on these findings, it was hypothesized that GA treatment might be accompanied by alterations in *PPAR*-related factors and the expression of genes involved in adipogenesis and lipid metabolism. This possibility was subsequently examined through intracellular lipid accumulation assays and the analysis of selected gene and protein expression levels in 3T3-L1 cells.

### 2.2. Ga Reduces Intracellular Lipid Accumulation During Adipocyte Differentiation

**Figure 2:**
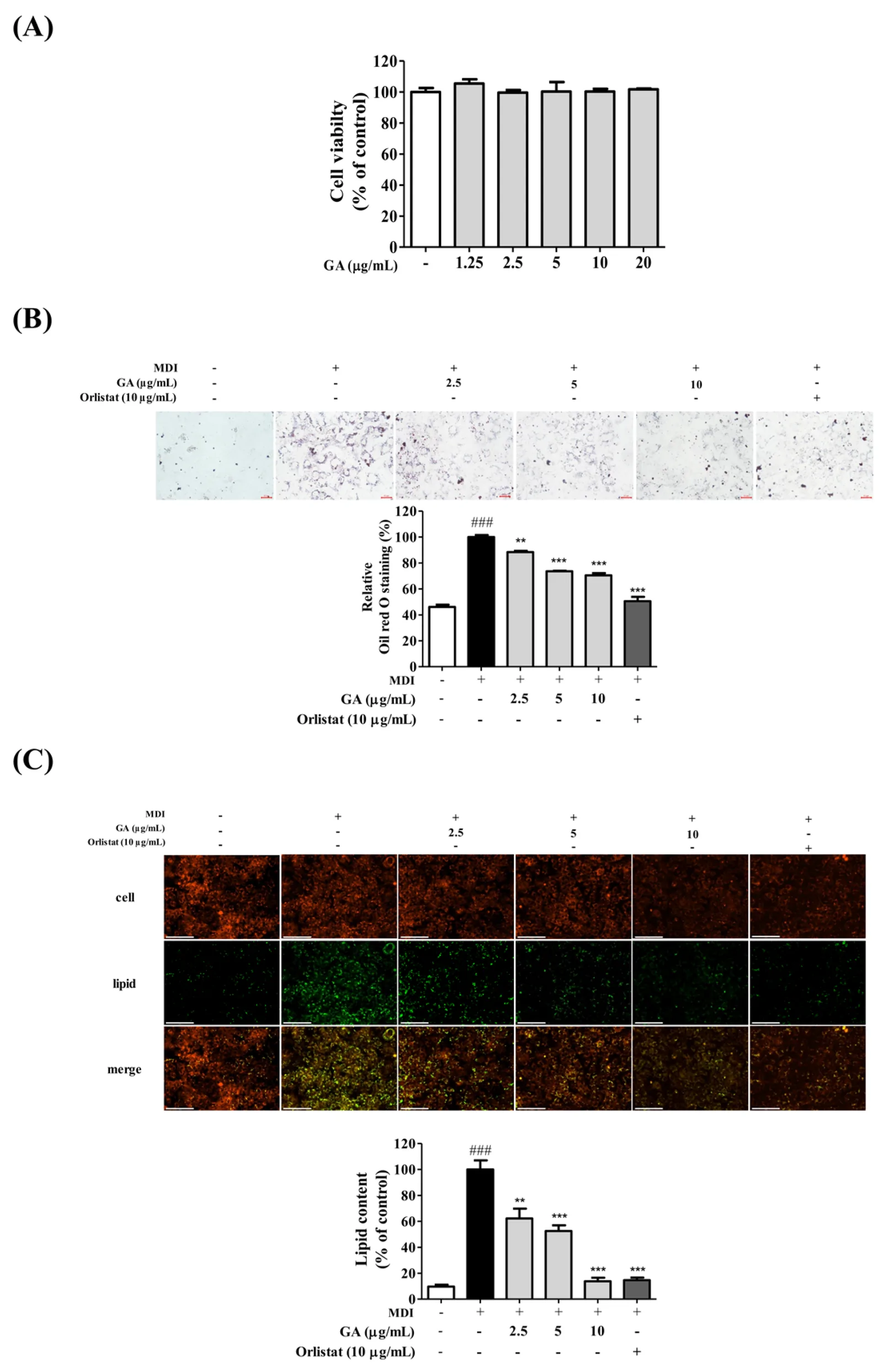
Inhibitory effects of GA on adipocyte differentiation and lipid accumulation in MDI-induced 3T3-L1 cells. (**A**) Cytotoxicity assessment of GA in 3T3-L1 preadipocytes by MTT assay after 24 h treatment. (**B**) Oil Red O staining showing intracellular lipid droplet accumulation and corresponding quantitative analysis. (**C**) AdipoRed fluorescence staining showing neutral lipid accumulation and ImageJ-based quantification of fluorescence intensity. Red and green fluorescence represent cellular staining and neutral lipid accumulation, respectively, while the merged images show both fluorescence signals. GA treatment reduced lipid accumulation in a concentration-dependent manner without affecting cell viability. Data are expressed as mean ± SD (*n* = 3). ### *p* < 0.001 versus the normal control group; ** *p* < 0.01, and *** *p* < 0.001 versus the MDI-treated group.

The effects of GA on 3T3-L1 preadipocyte metabolic viability were first evaluated using an MTT assay after 24 h of treatment (Figure 2A). GA concentrations ranging from 1.25 to 20 μg/mL did not significantly reduce MTT-derived viability measurements compared with the control group, with all treatment groups maintaining values above 90%. Based on these short-term observations, GA concentrations of 2.5, 5, and 10 μg/mL were selected for subsequent experiments. The effects of GA on adipocyte differentiation and intracellular lipid accumulation were subsequently assessed by Oil Red O and AdipoRed staining (Figure 2B,C). MDI-induced differentiation markedly increased lipid droplet formation and neutral lipid accumulation compared with the normal control group. In contrast, GA treatment reduced intracellular lipid accumulation in a concentration-dependent manner. Microscopic observation of Oil Red O-stained cells revealed extensive lipid droplet formation in the MDI-treated group, whereas lipid deposition was progressively reduced following GA treatment. Quantitative analysis further demonstrated significant reductions in lipid accumulation in the 5 and 10 μg/mL GA-treated groups, with the strongest inhibitory effect observed at 10 μg/mL. Consistent with the Oil Red O results, AdipoRed fluorescence imaging demonstrated increased neutral lipid accumulation in MDI-induced adipocytes, whereas GA treatment markedly reduced fluorescence intensity and intracellular lipid deposition. Quantitative ImageJ (version 1.54g; National Institutes of Health, Bethesda, MD, USA) analysis confirmed a concentration-dependent reduction in lipid-associated fluorescence signals, with the 10 μg/mL GA-treated group exhibiting a response comparable to that of the orlistat-treated positive control group. Collectively, these findings indicate that GA treatment was associated with reduced intracellular lipid accumulation during adipocyte differentiation.

### 2.3. Ga Regulates Cell Cycle Progression and Restores Mitochondrial Function During Adipocyte Differentiation

**Figure 3:**
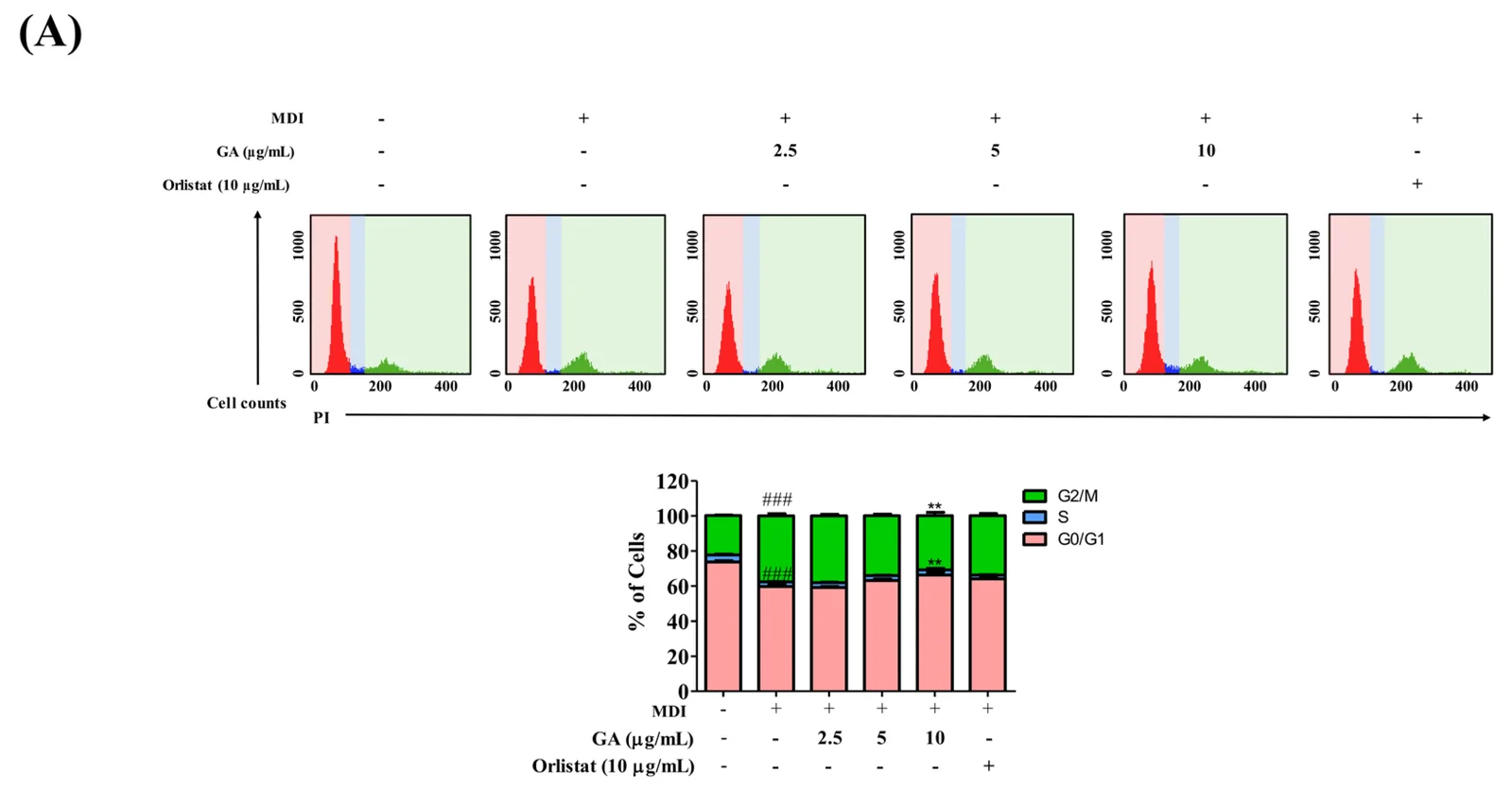

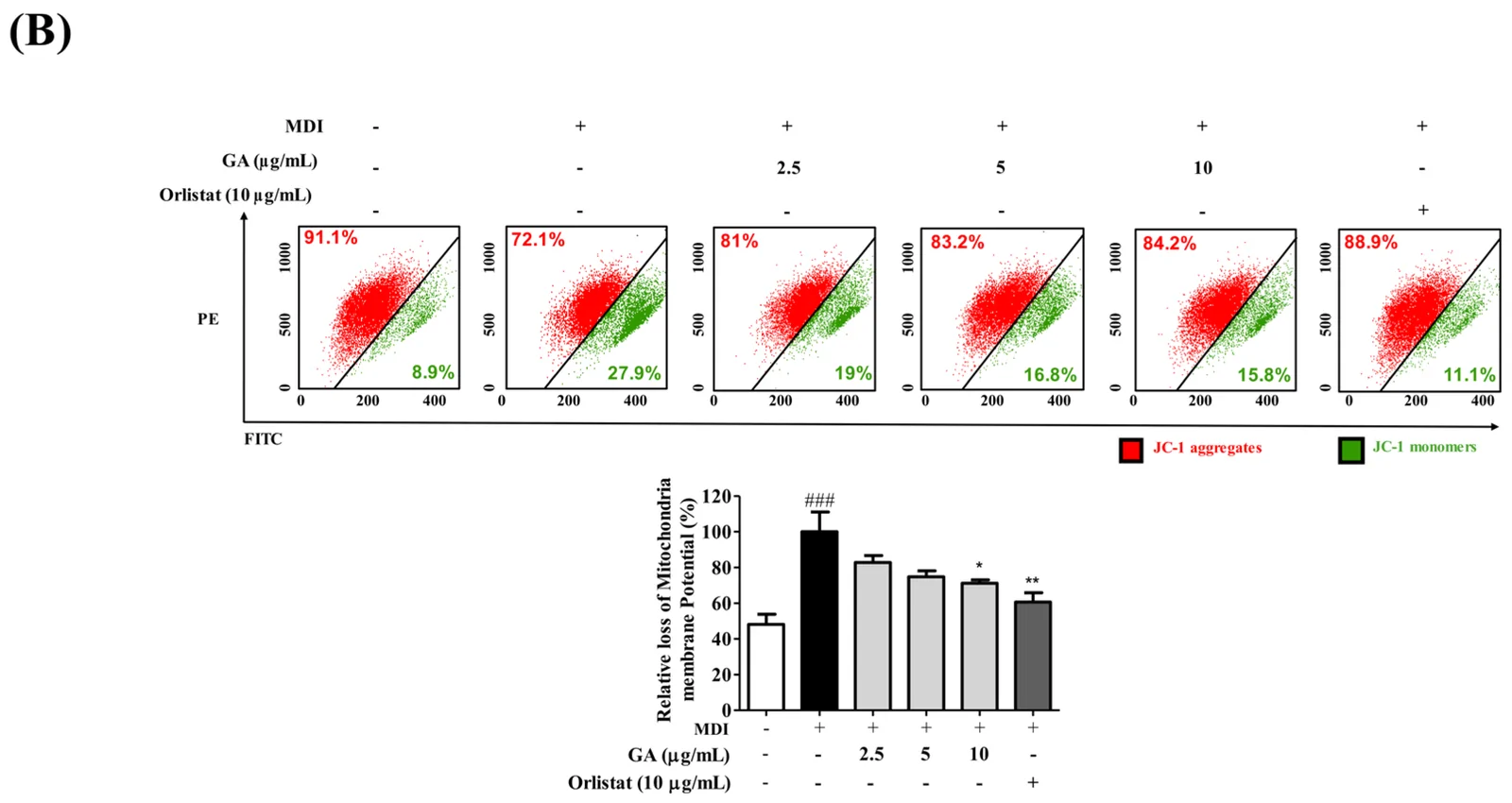
Regulatory effects of GA on cell cycle progression and mitochondrial function during adipocyte differentiation. (**A**) Flow cytometric analysis of cell cycle distribution in 3T3-L1 cells following 48 h of treatment with MDI and GA or orlistat during the initial differentiation induction period. GA treatment was associated with an increased proportion of cells in the G0/G1 phase and a decreased G2/M phase population compared with the MDI-treated group. (**B**) JC-1 staining and flow cytometric analysis of mitochondrial membrane potential. GA attenuated the MDI-induced loss of mitochondrial membrane potential by increasing the proportion of JC-1 aggregates and decreasing the proportion of JC-1 monomers. Data are expressed as mean ± SD (*n* = 3). ### *p* < 0.001 versus the normal control group; * *p* < 0.05, ** *p* < 0.01, and ### *p* < 0.001 versus the MDI-treated group.

To evaluate changes in cell cycle distribution during the initial phase of adipocyte differentiation, 3T3-L1 cells were analyzed 48 h after MDI induction using PI staining and flow cytometry (Figure 3A). Compared with the normal control group, MDI treatment decreased the proportion of cells in the G0/G1 phase from 73.72% to 59.69% and increased the G2/M phase population from 22.46% to 37.65%, whereas the S phase population decreased from 4.02% to 2.74%. GA treatment was associated with partial changes in these MDI-induced cell cycle distributions. Treatment with 5 and 10 μg/mL GA increased the proportion of cells in the G0/G1 phase to 63.16% and 66.33%, respectively, while reducing the G2/M phase population to 34.15% and 30.89%, respectively. Similar changes were observed in the orlistat-treated group. The effects of GA on mitochondrial activity during adipocyte differentiation were further evaluated using JC-1 staining and flow cytometric analysis (Figure 3B). MDI treatment markedly increased the proportion of JC-1 monomers and decreased the proportion of JC-1 aggregates compared with the normal control group, indicating a reduction in MMP. In contrast, GA treatment increased the proportion of JC-1 aggregates while reducing the monomer population relative to the MDI-treated group. Quantitative analysis revealed that the MDI-induced loss of MMP was significantly attenuated in the 10 μg/mL GA-treated group, and a similar trend was observed in the orlistat-treated positive control group. These findings suggest that GA may alleviate mitochondrial dysfunction associated with adipocyte differentiation and contribute to the maintenance of mitochondrial activity.

### 2.4. Ga Modulates the Expression of Adipogenic and Lipid Metabolism-Related Genes

**Figure 4:**
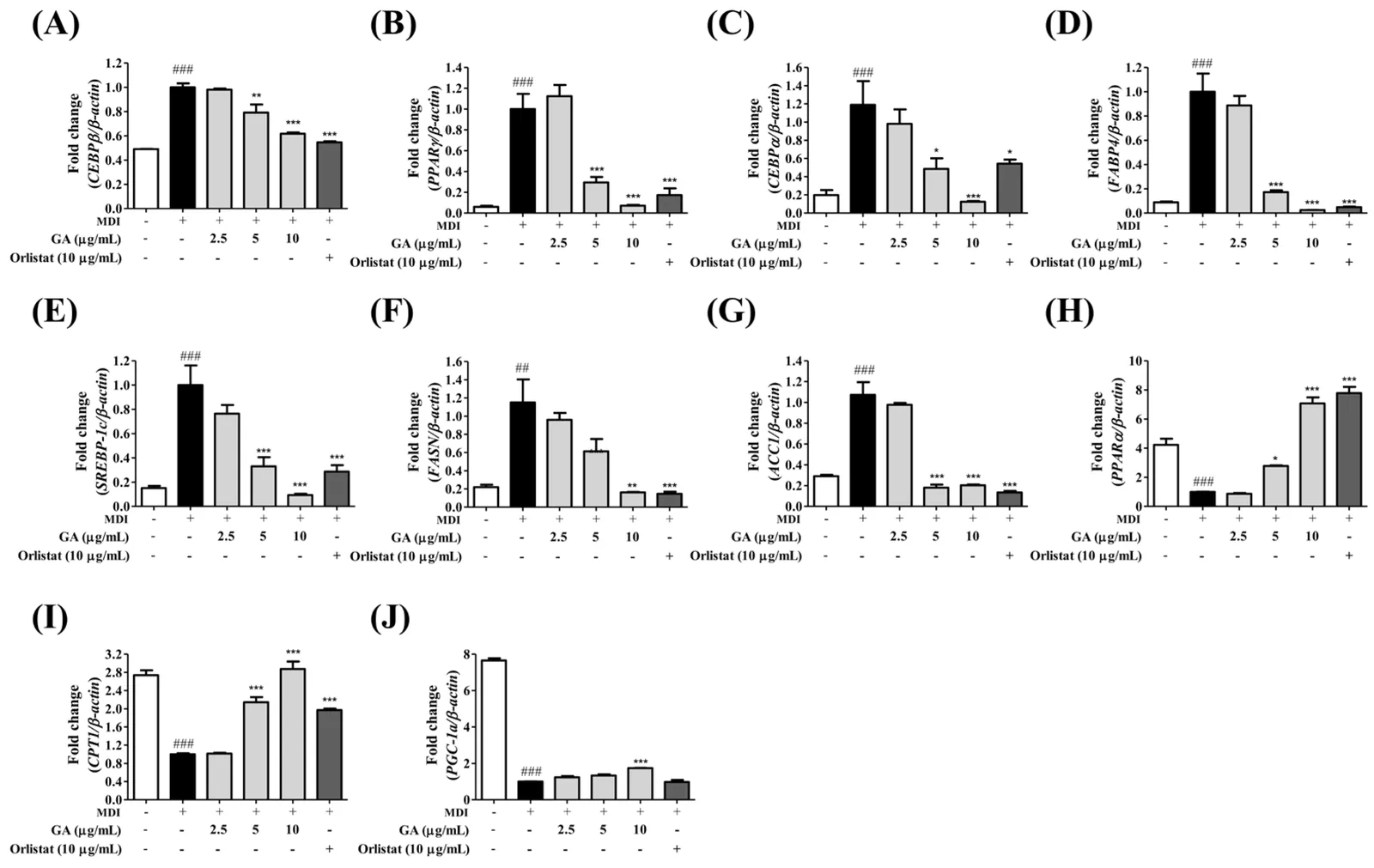
Regulation of adipogenic, lipogenic, and energy metabolism-related gene expression by GA in MDI-induced 3T3-L1 adipocytes. Relative mRNA expression levels were quantified by RT-qPCR following treatment with GA (2.5, 5, and 10 μg/mL) or orlistat (10 μg/mL). GA suppressed the expression of adipogenic markers (**A**) *Cebpb*, (**B**) *Pparg*, (**C**) *Cebpa*, *and* (**D**) *Fabp4,* and lipogenesis-related genes (**E**) *Srebp1c*, (**F**) *Fasn*, and (**G**) *Acaca*, while increasing the expression of genes associated with fatty acid oxidation and mitochondrial energy metabolism (**H**) *Ppara*, (**I**) *Cpt1*, and (**J**) *Ppargc1a*. Relative gene expression levels were normalized to *β-actin*. Data are expressed as mean ± SD (*n* = 3). ## *p* < 0.01, ### *p* < 0.001 versus the normal control group; * *p* < 0.05, ** *p* < 0.01, and *** *p* < 0.001 versus the MDI-treated group.

To investigate the molecular mechanisms underlying the anti-obesity effects of GA, the mRNA expression levels of genes associated with adipogenesis, lipogenesis, and energy metabolism were analyzed by RT-qPCR (Figure 4). MDI treatment significantly increased the expression of adipogenic markers, including *Cebpb*, *Pparg*, *Cebpa*, and *Fabp4*, whereas GA treatment suppressed their expression compared with the MDI-treated group. Expression of *Cebpb* was reduced by 20.8% and 38.3% in the 5 and 10 μg/mL GA-treated groups, respectively. Similarly, *PPARγ* expression was reduced by 70.6% and 93.0%, whereas *Cebpa* expression was decreased by 44.7% and 87.4% in the 5 and 10 μg/mL treatment groups, respectively. *Fabp4* expression was also markedly suppressed, showing reductions of 82.9% and 97.6% following treatment with 5 and 10 μg/mL GA, respectively (Figure 4A–D). The expression of lipogenesis-related genes was similarly affected by GA treatment. Compared with the MDI-treated group, *Srebp1c* expression was reduced by 67.0% and 90.5% in the 5 and 10 μg/mL GA-treated groups, respectively. *Fasn* expression showed a significant reduction in the 10 μg/mL treatment group, whereas a decreasing trend was observed at 5 μg/mL. *Acaca* expression was significantly decreased by 81.8% and 79.9% in the 5 and 10 μg/mL treatment groups, respectively (Figure 4E–G). In contrast, GA increased the expression of genes involved in energy metabolism and fatty acid oxidation. Relative to the MDI-treated group, *Ppara* expression increased 2.8-fold and 7.2-fold in the 5 and 10 μg/mL GA-treated groups, respectively. *Cpt1* expression was also elevated by approximately 2.1-fold and 2.9-fold following treatment with 5 and 10 μg/mL GA, respectively. In addition, *Ppargc1a* expression was significantly increased in the 10 μg/mL treatment group, while a non-significant increasing trend was observed at 5 μg/mL (Figure 4H–J). Similar expression patterns were observed in the orlistat-treated positive control group. Collectively, these findings indicate that GA suppresses adipogenic and lipogenic gene expression while promoting the expression of genes associated with energy metabolism during adipocyte differentiation.

### 2.5. Ga Treatment Is Associated with Changes in Ampk Phosphorylation and Adipogenic Protein Expression

**Figure 5:**
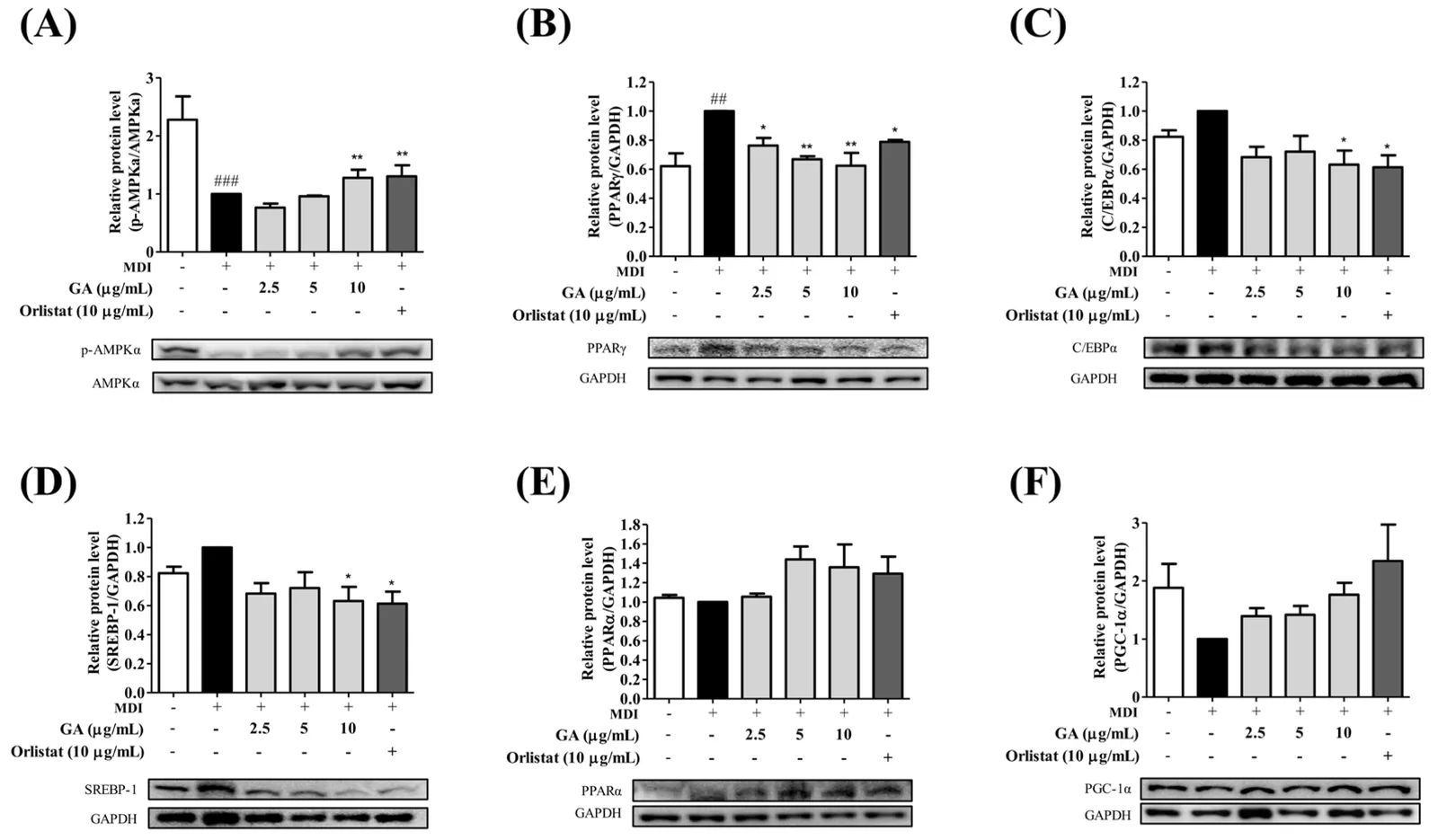
Regulation of AMPK signaling, adipogenic factors, and mitochondrial energy metabolism-related proteins by GA in MDI-induced 3T3-L1 adipocytes. Western blot analysis was performed to evaluate protein expression levels following treatment with GA (2.5, 5, and 10 μg/mL) or orlistat (10 μg/mL). (**A**) p-AMPKα/AMPKα ratio, (**B**) PPARγ, (**C**) C/EBPα, (**D**) SREBP-1, (**E**) PPARα, and (**F**) PGC-1α. GA restored the reduced p-AMPKα/AMPKα ratio and suppressed the expression of adipogenic and lipogenic proteins (PPARγ, C/EBPα, and SREBP-1), while increasing proteins associated with fatty acid oxidation and mitochondrial energy metabolism (PPARα and PGC-1α). Representative immunoblots and corresponding densitometric analyses are shown. Protein expression levels were normalized to GAPDH. Data are expressed as mean ± SD (*n* = 3). ## *p* < 0.01, ### *p* < 0.001 versus the normal control group; * *p* < 0.05, and ** *p* < 0.01versus the MDI-treated group.

To determine whether the transcriptional changes observed following GA treatment were accompanied by alterations in AMPK-related signaling, protein expression levels of p-AMPKα, AMPKα, adipogenesis-related factors, and energy metabolism-related factors were analyzed by Western blot (Figure 5). Compared with the normal control group, MDI treatment reduced the p-AMPKα/AMPKα ratio. Following GA treatment, the p-AMPKα/AMPKα ratio was lower than that of the MDI-treated group at 2.5 μg/mL and remained close to the MDI level at 5 μg/mL, whereas an increase was observed at 10 μg/mL (1.28-fold relative to the MDI-treated group). These findings suggest that increased AMPKα phosphorylation was observed primarily at the highest GA concentration, rather than consistently across the tested concentrations. The orlistat-treated group exhibited a 1.30-fold ratio relative to the MDI-treated group (Figure 5A), whereas total AMPKα expression remained relatively unchanged among the experimental groups. The effects of GA on adipogenesis-related proteins were subsequently examined. Compared with the MDI-treated group, PPARγ expression was reduced to 0.75-, 0.77-, and 0.79-fold in the 2.5, 5, and 10 μg/mL GA-treated groups, respectively, with significant reductions observed at 5 and 10 μg/mL (Figure 5B). Similarly, C/EBPα expression was reduced to 0.95-, 0.82-, and 0.84-fold in the respective treatment groups (Figure 5C). The lipogenic transcription factor SREBP-1 was also suppressed following GA treatment, exhibiting expression levels of 0.68-, 0.72-, and 0.63-fold relative to the MDI-treated group in the 2.5, 5, and 10 μg/mL treatment groups, respectively, with the greatest reduction observed at 10 μg/mL (Figure 5D). In contrast, GA increased the expression of proteins associated with energy metabolism. Relative to the MDI-treated group, PPARα expression increased to 1.05-, 1.44-, and 1.36-fold in the 2.5, 5, and 10 μg/mL GA-treated groups, respectively (Figure 5E). Likewise, PGC-1α expression increased to 1.39-, 1.42-, and 1.86-fold in the corresponding treatment groups, with the highest expression observed in the 10 μg/mL treatment group (Figure 5F). Similar expression patterns were observed in the orlistat-treated positive control group. Collectively, these findings indicate that GA treatment was associated with increased AMPKα phosphorylation at 10 μg/mL, reduced expression of adipogenic and lipogenic proteins, and increased expression of proteins associated with energy metabolism during adipocyte differentiation.

### 2.6. Computational Assessment of Ga Docking Configurations and Molecular Dynamics in Pparα and Pparγ Models

**Figure 6:**
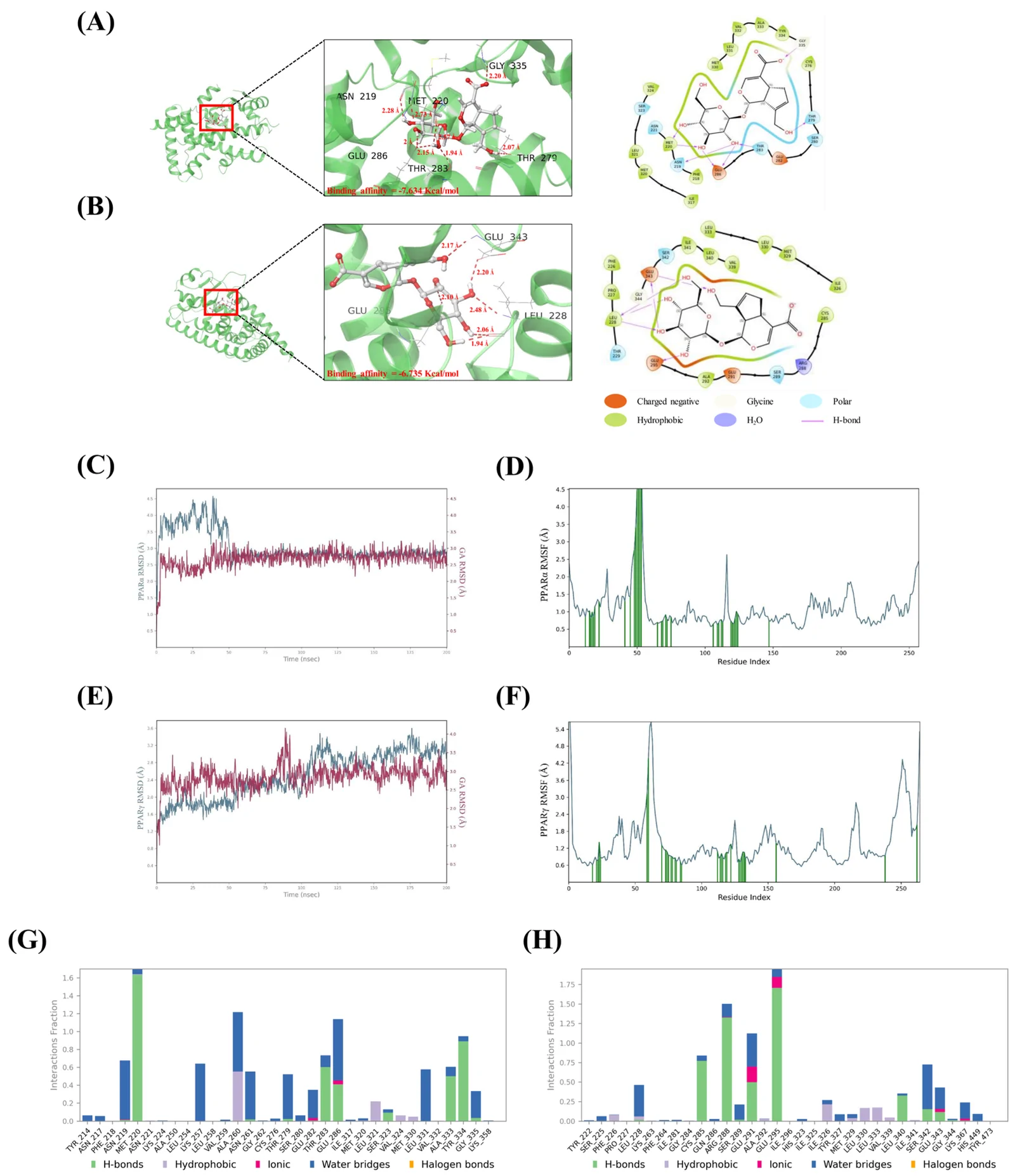
Computational evaluation of GA interactions with PPARα and PPARγ through molecular docking and MD simulations. (**A**,**B**) Predicted binding modes and interaction profiles of GA within the ligand-binding domains of PPARα and PPARγ, respectively. (**C**–**F**) RMSD and RMSF analyses of the GA–PPARα and GA–PPARγ complexes during 200 ns MD simulations, demonstrating the dynamic stability of the protein–ligand systems. (**G**,**H**) Interaction fraction analyses showing the persistence of hydrogen bonds, hydrophobic contacts, ionic interactions, and water-mediated bridges between GA and key amino acid residues throughout the simulation period. The results support stable interactions of GA with both PPARα and PPARγ.

Based on the network pharmacology, RT-qPCR, and Western blot results, molecular docking analysis was performed to evaluate the potential interactions of GA with PPARα and PPARγ (Figure 6A,B). The calculated docking scores for the selected poses were −7.634 kcal/mol for PPARα and −6.735 kcal/mol for PPARγ. The predicted docking poses showed potential hydrogen-bonding and hydrophobic contacts with residues surrounding the modeled binding regions. These scores represent computational estimates and do not directly establish binding affinity or specificity. To further examine the conformational behavior of the modeled systems, 200 ns molecular dynamics (MD) simulations were performed for the GA–PPARα (Figure 6C,D) and GA–PPARγ complexes (Figure 6E,F). RMSD analysis showed initial fluctuations followed by relatively stable trajectories during the later stages of the simulations, particularly after approximately 50 ns. RMSF analysis indicated relatively limited fluctuations in most protein regions, with greater flexibility observed in certain loop regions. Interaction fraction analysis further revealed recurring contacts between GA and several amino acid residues within the simulated systems (Figure 6G,H). Consistent with the molecular docking predictions, hydrogen-bonding and water-mediated interactions were observed involving MET 220, THR 283, GLU 286, TYR 334, and GLY 335 in the PPARα complex (Figure 6G). Similarly, recurring contacts involving GLU 295, ARG 288, LEU 228, and GLU 343, were identified in the modeled GA–PPARγ system, with hydrogen-bonding and water-mediated bridges among the observed interaction types (Figure 6H). The occurrence of these contacts during the simulation period was consistent with the relatively stable conformational trajectories observed in the modeled systems. Collectively, these computational results provided information on possible docking configurations and conformational characteristics of GA within the modeled PPARα and PPARγ systems. However, these observations do not establish direct molecular binding or demonstrate the functional involvement of PPAR signaling under cellular conditions.

## 3. Discussion

Obesity is a chronic metabolic disorder characterized by excessive adipose tissue accumulation and is associated with numerous complications, including insulin resistance, cardiovascular disease, and nonalcoholic fatty liver disease. Since obesity arises from complex interactions between adipogenesis, lipid metabolism, and energy homeostasis, there is significant interest in identifying bioactive compounds capable of modulating these processes. In the present study, the cellular responses associated with geniposidic acid (GA) treatment were investigated using 3T3-L1 adipocytes, supported by network pharmacology and computational analyses. The identification of 57 overlapping GA-associated and obesity-related candidate targets suggested potential involvement of metabolic pathways, including PPAR signaling. However, these computationally identified associations should be interpreted as hypothesis-generating findings rather than evidence that GA directly regulates the predicted targets. In particular, the expanded protein–protein interaction network incorporated functionally associated proteins, and its connectivity does not establish direct molecular interactions between GA and individual proteins.

Adipocyte differentiation is regulated through the coordinated and sequential activity of transcription factors, including *Cebpb*, *Pparg*, and *Cebpa*, which play important roles in adipocyte maturation and lipid accumulation [24]. These transcription factors stimulate the expression of adipocyte-specific genes, including *Fabp4*, thereby promoting lipid droplet formation and adipocyte maturation [25,26]. In the present study, the reduction in intracellular lipid accumulation following GA treatment was accompanied by decreased mRNA expression of adipogenesis-related genes, including *Cebpb*, *Pparg*, *Cebpa*, and *Fabp4*. These findings are consistent with alterations in the adipogenic transcriptional program, suggesting that GA treatment may attenuate cellular processes associated with adipocyte differentiation and lipid storage. In particular, the concurrent reduction in *Cebpb* expression and downstream adipogenic markers suggests that GA may influence multiple components of the adipogenic regulatory network, although these observations do not establish direct inhibition of the transcriptional cascade or identify the specific stage at which GA exerts its effects [27]. Previous research has reported that GA improved serum lipid profiles and attenuated hepatic lipid accumulation in high-fat-diet-induced mice [22]. These findings are consistent with the reduced intracellular lipid accumulation observed in GA-treated 3T3-L1 adipocytes in the present study. However, differences in experimental models and measured outcomes should be considered, and these observations do not establish a shared molecular mechanism.

Orlistat was employed as a positive control for intracellular lipid accumulation assays based on its previous use in 3T3-L1 adipocyte studies. The concentration used in the present study (10 μg/mL, approximately 20 μM) was consistent with those reported in previous in vitro investigations [28,29]. However, the primary clinical action of orlistat involves the inhibition of gastrointestinal lipases, which is distinct from the cellular signaling processes examined in this study. Accordingly, orlistat was used to provide a comparative reference for the observed cellular responses rather than to establish a shared mechanism of action with GA. Although changes in selected gene and protein expression levels were observed in both treatment groups, these findings do not demonstrate that GA and orlistat act through the same molecular pathways. Furthermore, the concentration used in this cell-based study was not intended to reproduce clinically achieved systemic exposure.

Cell cycle progression is another important process associated with adipocyte differentiation. When growth-arrested preadipocytes are exposed to differentiation stimuli, they re-enter the cell cycle and undergo mitotic clonal expansion (MCE), which is closely associated with the early stages of adipogenesis [30,31]. MCE is also associated with *Cebpb* induction and the subsequent expression of adipogenic transcription factors, including *Pparg* and *Cebpa*. In the present study, cell cycle distribution was evaluated 48 h after MDI induction, corresponding to the end of the initial differentiation induction period. This time point was selected to examine changes in cell cycle distribution following exposure to differentiation stimuli, consistent with previous studies using a similar experimental approach [32,33]. Compared with the MDI-treated group, GA treatment was associated with an increased proportion of cells in the G0/G1 phase and a decreased proportion in the G2/M phase. These findings suggest that GA treatment may be associated with alterations in cell cycle regulation during early adipocyte differentiation. However, the observed changes cannot be attributed specifically to MCE inhibition, as cell cycle distribution was assessed at a single 48 h time point without direct evaluation of DNA synthesis or cell proliferation. Therefore, these changes may reflect broader effects on proliferation- or differentiation-associated cell cycle regulation. Together with the observed alterations in the expression of *Cebpb*, *Pparg*, and *Cebpa*, these findings suggest a potential association between GA treatment and cellular processes involved in adipocyte differentiation, although the specific involvement of MCE remains to be determined.

In addition, the regulation of adipogenic transcription factors, lipid synthesis is an important metabolic process during adipocyte differentiation. *Srebp1c* acts as a major transcriptional regulator of fatty acid synthesis, while *Fasn* and *Acaca* are key downstream enzymes involved in de novo lipid synthesis [31]. The reduced expression of lipogenesis-related genes following GA treatment was consistent with the observed decrease in intracellular lipid accumulation, suggesting that changes in lipid synthesis-related regulation may contribute to the cellular responses associated with GA treatment. Notably, ACC1 is a known downstream target of AMPK and is negatively regulated through AMPK-mediated phosphorylation [34]. However, the reduction in Acaca mRNA expression observed in the present study does not directly demonstrate AMPK-mediated ACC1 phosphorylation or enzymatic inhibition.

AMPK functions as a central cellular energy sensor that regulates lipid metabolism and energy homeostasis. Activation of AMPK suppresses fatty acid synthesis while promoting fatty acid oxidation, and impaired AMPK signaling is frequently associated with obesity and insulin resistance [35]. In the present study, an increase in the p-AMPKα/AMPKα ratio relative to the MDI-treated group was observed at 10 μg/mL GA, whereas lower concentrations did not exhibit a consistent increase. Previous studies have reported that AMPK phosphorylation responses in 3T3-L1 cells may differ depending on treatment concentration. Chen et al. (2018) demonstrated that metformin increased AMPK phosphorylation at higher concentrations, whereas lower concentrations did not produce significant changes [36]. Although the cellular responses to metformin and GA are not directly comparable, these findings suggest that AMPK phosphorylation may exhibit different sensitivities to treatment concentration. In the present study, the reduction in adipogenic and lipogenic gene expression at lower GA concentrations was not consistently accompanied by increased AMPKα phosphorylation, suggesting that these cellular responses may not be solely associated with AMPK activation. Thus, the observed discrepancy may reflect differences in the concentration sensitivity of AMPK phosphorylation and adipogenesis-related responses, potentially involving AMPK-independent regulatory processes. However, the specific mechanisms underlying these differences remain to be determined. In addition to changes in adipogenic and lipogenic factors, GA treatment was associated with alterations in markers related to mitochondrial energy metabolism. *Ppara* is a nuclear receptor that promotes fatty acid β-oxidation, *Cpt1* regulates mitochondrial fatty acid transport, and *Ppargc1a* functions as a key regulator of mitochondrial biogenesis and oxidative metabolism [37]. The increased expression of genes associated with these processes, together with attenuation of the MDI-associated reduction in mitochondrial membrane potential, suggests that GA treatment may influence cellular processes involved in mitochondrial metabolic regulation. Mitochondrial dysfunction has been implicated in adipocyte metabolic dysregulation and obesity-related inflammation [38,39], while PGC-1α-associated pathways are involved in mitochondrial metabolic adaptations [40,41]. Nevertheless, restoration of mitochondrial membrane potential does not independently demonstrate increased mitochondrial biogenesis, fatty acid oxidation, or energy expenditure. Therefore, the observed findings should be interpreted as changes in mitochondrial function-related indicators rather than direct evidence of enhanced mitochondrial oxidative metabolism.

To further explore the potential involvement of PPAR-related factors identified through network pharmacology analysis, molecular docking and molecular dynamics simulations were performed using computational models of PPARα and PPARγ. Docking analysis generated predicted molecular configurations with calculated scores of −7.634 and −6.735 kcal/mol for PPARα and PPARγ, respectively, suggesting possible interactions within the modeled binding regions. Subsequent 200 ns MD simulations showed relatively stable conformational trajectories, accompanied by recurring hydrogen-bonding and water-mediated contacts involving residues within the predicted binding regions. These observations were consistent with the conformational stability of the modeled systems under the selected simulation conditions. However, docking scores represent computational estimates rather than direct measurements of binding affinity or specificity, and the stability observed during MD simulations does not establish receptor binding or functional activity under experimental conditions. Although GA treatment was associated with alterations in PPAR-related gene and protein expression, the relationship between these cellular responses and the computational predictions remains uncertain. Therefore, these findings provide exploratory structural information regarding possible molecular configurations of GA within PPARα and PPARγ models, rather than direct evidence of receptor engagement or modulation of PPAR signaling activity.

Several limitations should be acknowledged. First, the present study was conducted using a 3T3-L1 adipocyte model and therefore may not fully reflect the complexity of obesity in vivo. Second, the network pharmacology analysis relied on candidate targets retrieved from multiple public databases, which differ in their prediction methods, data sources, and scoring systems. As no additional user-defined score-based filtering was applied during target retrieval, some candidate targets may represent low-confidence or false-positive associations. Furthermore, the inclusion of functionally associated proteins during PPI network expansion may have introduced indirect associations that do not necessarily represent direct molecular interactions with GA. Species-related differences should also be considered when interpreting human protein annotations in the context of murine 3T3-L1 cells. Although subsequent cellular experiments showed alterations in lipid accumulation and the expression of selected adipogenic and metabolic factors, these observations do not independently validate the predicted molecular targets. Third, the absence of AMPK inhibition or knockdown experiments prevents determination of whether AMPK signaling is causally required for the observed cellular responses. Similarly, molecular docking and molecular dynamics simulations provide computational predictions of potential protein–ligand interactions but do not directly establish receptor binding or activation under physiological conditions. Fourth, the 24 h MTT assay does not exclude delayed cytotoxicity, changes in cell number, or metabolic alterations during the 10-day differentiation period. Consequently, the observed decrease in intracellular lipid accumulation cannot be conclusively distinguished from potential effects of prolonged treatment on cell viability or cellular metabolism. Further studies involving direct target validation, functional pathway analyses, long-term viability assessments, and in vivo investigations are needed to clarify the biological relevance of the observed GA-associated responses.

## 4. Materials & Methods

### 4.1. Cell Culture and Treatment

3T3-L1 pre-adipocyte cells were obtained from the Korean Cell Line Bank (Seoul, Republic of Korea) and maintained in DMEM supplemented with 10% bovine calf serum (BCS; HyClone, Carlsbad, CA, USA) and 1% penicillin–streptomycin (Gibco, Grand Island, NY, USA). For adipocyte differentiation, confluent cells were cultured in differentiation medium containing 10% fetal bovine serum (FBS), 0.5 mM IBMX, 1 μM dexamethasone, and 1 μg/mL insulin for 2 days. The medium was subsequently replaced with DMEM containing 10% FBS and 1 μg/mL insulin for 2 days, followed by DMEM supplemented with 10% FBS alone, with medium changes every 2 days until day 10. Geniposidic acid (GA; purity ≥98%, ChemFaces, Wuhan, China) and orlistat (Sigma-Aldrich, St. Louis, MO, USA) were dissolved in dimethyl sulfoxide (DMSO) and administered throughout the differentiation period. Orlistat was used at 10 μg/mL (approximately 20 μM) as a positive control for evaluating intracellular lipid accumulation, consistent with concentrations previously employed in 3T3-L1 adipocyte studies [28,29]. In subsequent cellular and molecular analyses, the orlistat-treated group was included as a pharmacological comparator rather than a pathway-specific positive control for AMPK activation or PPAR signaling. The final concentration of DMSO did not exceed 0.1% (*v*/*v*).

### 4.2. Cell Viability

3T3-L1 cells were seeded into a 96-well plate at a density of 2 × 10^3^ cells per well. After 24 h of culture, the cells were treated with GA at various concentrations ranging from 1.25 to 20 μg/mL. After 24 h of treatment, the samples were incubated with MTT reagent for 4 h, washed with warm PBS, dissolved in DMSO, and the absorbance was measured. Cell viability was measured at 540 nm using a microplate reader (Infinite 200; TECAN, Zürich, Switzerland).

### 4.3. Compound and Disease Target Prediction

Potential targets of GA were collected from GeneCards (https://www.genecards.org/; accessed on 14 January 2026), STITCH (https://stitch.embl.de/; accessed on 14 January 2026), SwissTargetPrediction (https://www.swisstargetprediction.ch/; accessed on 14 January 2026), and TargetNet (https://targetnet.scbdd.com/home/index/; accessed on 14 January 2026). All target searches were conducted using Homo sapiens (human) as the reference species. Target retrieval was performed using the default search settings of each database without applying additional user-defined scores or confidence thresholds. The retrieved targets were standardized and converted into official gene symbols using the UniProt ID mapping tool, and duplicate entries were removed. Obesity-associated targets were obtained from GeneCards and DisGeNET (https://disgenet.com/; accessed on 14 January 2026), using Homo sapiens as the reference species and without additional user-defined score-based filtering. The retrieved GeneCards obesity-related dataset comprised protein-coding genes. Following the removal of duplicate entries, followed by the removal of redundant entries. Common targets between GA and obesity were identified using the Bioinformatics platform (https://www.bioinformatics.com.cn/en/; accessed on 14 January 2026), where a Venn diagram was generated for analysis. The overlapping targets were subsequently imported into Cytoscape (version 3.10.3) for network construction and visualization.

### 4.4. Gene Ontology (Go) and Kegg Pathway Enrichment Analysis

Functional annotation enrichment analysis was conducted using the Enrichr platform (https://maayanlab.cloud/Enrichr/; accessed on 14 January 2026) to investigate the biological functions and signaling pathways associated with the 57 overlapping targets identified between GA and obesity-related genes. GO enrichment analysis was conducted using the GO Biological Process 2025, GO Cellular Component 2025, and GO Molecular Function 2025 libraries, together with Kyoto Encyclopedia of Genes and Genomes (KEGG) pathway analysis. No custom background gene set was specified, and the analysis was performed using the default settings of Enrichr. Statistical significance was evaluated using the multiple-testing-adjusted *p*-values provided by Enrichr, with adjusted *p*-values below 0.05 considered statistically significant. For visualization, the top 10 GO terms within each category and the top 20 KEGG pathways were selected based on their nominal *p*-values and visualized using the Bioinformatics platform (https://www.bioinformatics.com.cn/en/; accessed on 14 January 2026).

### 4.5. Protein–Protein Interaction (Ppi) Network Analysis

Protein–protein interaction (PPI) analysis was conducted using the STRING database (https://string-db.org/; accessed on 15 January 2026) with a confidence score threshold of 0.400. Each of the 57 overlapping targets was individually queried in STRING to retrieve functionally associated protein partners. The resulting interaction datasets were integrated and merged using Cytoscape (version 3.10.3) to construct an expanded PPI network. Network topology was evaluated using the Network Analyzer tool, and node degree was used to assess connectivity. Based on the average degree value of the expanded network, nodes with degree values ≥10 were selected for further analysis and visualization. A drug–target–pathway network was subsequently established by integrating the overlapping targets and enriched KEGG pathways. The expanded PPI network was used to explore functional associations among candidate targets and related proteins rather than to identify direct molecular interactions with GA.

### 4.6. Adipored Staining

Differentiated 3T3-L1 adipocytes were fixed with 4% formaldehyde on day 10 and stained using the AdipoRed assay reagent (Lonza, Basel, Switzerland) according to the manufacturer’s protocol. Fluorescence images were captured using an EVOS M7000 Imaging System (Thermo Fisher Scientific, Waltham, MA, USA) at 40× magnification with a 150 μm scale bar, and adipocyte area (μm^2^) was quantified using ImageJ software (version 1.54g; National Institutes of Health, Bethesda, MD, USA).

### 4.7. Oil Red O Staining

Intracellular lipid accumulation was evaluated by Oil Red O staining. Differentiated 3T3-L1 adipocytes were fixed with 4% formaldehyde on day 10 and stained with freshly prepared Oil Red O solution for 30 min at room temperature. Representative images were acquired using a microscope-mounted digital camera system (E3ISPM03100KPA, ToupTek Photonics Co., Ltd., Hangzhou, China). For quantitative analysis, the retained dye was dissolved in isopropanol, and absorbance was measured at 520 nm.

### 4.8. Cell Cycle Assay

Cell cycle distribution was determined by flow cytometry. The analysis was conducted 48 h after MDI induction, corresponding to the end of the initial differentiation induction period before replacement with insulin-containing medium. This time point was selected to evaluate changes in cell cycle distribution following the initial exposure to differentiation stimuli, consistent with previous studies examining cell cycle alterations during early adipocyte differentiation [32,33]. After treatment with MDI and GA for 2 days, 3T3-L1 cells were fixed in 70% ethanol, stained with propidium iodide (PI)-labeled RNase A (Sigma-Aldrich), and analyzed using a BD Accuri C6 flow cytometer (BD Accuri C6, BD Bioscience, Franklin Lakes, NJ, USA).

### 4.9. Assessment of Mitochondrial Membrane Potential (Mmp)

Changes in mitochondrial membrane potential (MMP) were assessed using a JC-1 mitochondrial staining kit (Sigma-Aldrich, St. Louis, MO, USA). After treatment with GA, cells were incubated with JC-1 dye for 20 min and subsequently analyzed by flow cytometry. Valinomycin-treated cells were included as a positive control to verify JC-1 staining and mitochondrial membrane depolarization.

### 4.10. Quantitative Real-Time Pcr (Rt-Qpcr)

Total RNA was extracted from 3T3-L1 cells using TransZol Up reagent (TransGen Biotech, Beijing, China) according to the manufacturer’s instructions. RNA concentration was determined by spectrophotometric analysis (Thermo Fisher Scientific, Waltham, MA, USA). Complementary DNA (cDNA) was synthesized from 2 μg of total RNA using AccuPower^®^ RT PreMix (Bioneer, Daejeon, Republic of Korea). Quantitative real-time PCR (RT-qPCR) was subsequently performed using SYBR Green chemistry on a QuantStudio 3 system (Applied Biosystems, Waltham, MA, USA). The amplification conditions consisted of an initial denaturation at 95 °C for 3 min, followed by 40 cycles of denaturation at 95 °C for 30 s, annealing at 60 °C for 30 s, and extension at 72 °C for 30 s. Melting curve analysis was performed over a temperature range of 55–95 °C to assess amplification specificity and confirm the presence of a single amplification product. Relative gene expression levels were calculated using the 2^−ΔΔCt^ method and normalized to *β-actin*. Primer sequences used in this study are listed in Table 1.

### 4.11. Protein Expression Analysis via Western Blotting

Protein expression was analyzed by Western blot. Total protein extracted from 3T3-L1 cells using RIPA buffer (Sigma-Aldrich) was quantified by Bradford assay and separated by SDS-PAGE, followed by transfer to nitrocellulose membranes. After incubation with primary and secondary antibodies, protein bands were visualized using West-Q Pico chemiluminescent substrate (GenDEPOT, Katy, TX, USA) and an Alliance Mini HD9 imaging system (UVITEC, Cambridge, UK). Antibody information is summarized in Table 2.

### 4.12. Computational Docking Analysis

The binding interactions of GA with target proteins PPARα as well as PPARγ were investigated through molecular docking analysis using Maestro (Schrodinger, Inc., New York, NY, USA). The two-dimensional structure of GA was obtained from the PubChem database (https://pubchem.ncbi.nlm.nih.gov/; accessed on 30 January 2026). Furthermore, the crystal structures of PPARα (PDB: 3VI8) and PPARγ (PDB: 6MS7) were retrieved from the RCSB Protein Data Bank. Protein preparation was subsequently performed using the Protein Preparation Wizard in Maestro, which included tasks such as missing side-chain completion, hydrogen bond optimization, formal charge assignment, and removal of crystallographic water molecules. Ligand preparation was performed using Lig Prep under default settings, including Epik Classic mode, and optimization based on the OPLS4 force field. For identification of potential binding sites, Site Map analysis was subsequently applied to both protein structures and binding sites were determined based on site scores and volumes using default thresholds. Following SiteMap analysis, multiple predicted binding regions were considered for molecular docking. Receptor grids corresponding to the candidate sites were generated using the Receptor Grid Generation module in Maestro. For each grid, the center was manually defined by adjusting the X, Y, and Z coordinates, and the grid dimensions were adjusted to accommodate the selected binding region and GA. Separate docking calculations were performed for the candidate binding regions using Maestro’s Ligand Docking Wizard in standard precision (SP) mode. The resulting docking poses were compared based on their Glide docking scores, and the pose with the most favorable docking score was selected for subsequent structural analysis.

### 4.13. Molecular Dynamics (Md) Simulation

Molecular dynamics (MD) simulations of the PPARα–GA and PPARγ–GA complexes were performed using the Desmond package implemented in Maestro (Schrödinger, New York, NY, USA). The systems were prepared using the OPLS4 force field and solvated with the TIP3P water model in an orthorhombic simulation box with a 10 Å buffer distance. Counter ions and physiological salt concentrations were automatically added to neutralize the systems. Following system equilibration under the default protocol, production simulations were carried out for 200 ns. Simulation trajectories were analyzed using the Simulation Interaction Diagram tool to evaluate complex stability and protein–ligand interaction profiles.

### 4.14. Statistical Analysis

Data are presented as the mean ± standard deviation (SD). Statistical analyses were performed using GraphPad Prism version 5.0 (GraphPad Software, San Diego, CA, USA). Differences among groups were evaluated by one-way analysis of variance (ANOVA) followed by Tukey’s post hoc test. A *p*-value < 0.05 was considered statistically significant.

## 5. Conclusions

In conclusion, this study demonstrated that GA promotes mitochondrial energy metabolism during the process of adipocyte differentiation while simultaneously inhibiting adipogenesis and lipid synthesis. GA activated the AMPK signaling pathway, downregulated factors related to adipogenesis and lipid synthesis, and increased the expression of PPARα-related energy metabolism markers, ultimately reducing intracellular lipid accumulation. Furthermore, key obesity-related pathways were identified through network pharmacology analysis, and molecular docking and molecular dynamics simulations supported the possibility of GA interacting with PPARα and PPARγ. Collectively, these results suggest the potential of GA as a promising natural bioactive compound for the prevention or improvement of obesity-related metabolic disorders.

## Figures and Tables

**Table 1:** The primers we used in RT-qPCR.

Gene	Size (bp)	GenBank Accession No.	Primer Sequence (5′ → 3′)
*Cebpb*	119	NM_001287738	F: TCTACTACGAGCCCGACTGC R: GTAGGGGCTGAAGTCGATGG
*Pparg*	102	NM_011146	F: TCGCTGATGCACTGCCTATG R: GAGAGGTCCACAGAGCTGATT
*Cebpa*	87	NM_007678	F: GGTGGACAAGAACAGCAACG R: CGTTGTTTGGCTTTATCTCG
*Fabp4*	131	NM_024406	F: GCCCAACATGATCATCAGC R: TCACCTTCCTGTCGTCTGC
*Srebp1c*	73	NM_011480	F: TGTGGAGCTCAAAGACCTG R: TGTGCTGCAAGAAGCGGATG
*Fasn*	138	NM_007988	F: ACCCCAGCAGACACTGTAGC R: AAACCCAAGCATCATTTTCG
*Acaca*	138	NM_133360	F: AGAGAAAGTGCAGGCAGAGC R: TGAGCAGGAAGGACTTGACCC
*Ppara*	90	NM_011144	F: CGGGGAACTTAGAGGAGAGC R: TCTTGTTCCCGAACTTGACC
*Cpt1*	182	NM_013495	F: GTCAAGCCAGACGAAGAACA R: CGAGAAGACCTTGACCATAG
*Ppargc1a*	198	NM_008904	F: GGATGAATACCGCAAAGAGC R: GCGGTCTCTCAATTCTGTCC
*β-actin*	87	NM_007393	F: TGTCCACCTTCCAGCAGATGT R: AGCTCAGTAACAGTCCGCCTAGA

**Table 2:** The detailed information about antibodies used in this study.

Antibody	Catalog Number	Supplier	Dilution
p-AMPKa (Thr172)	2535	Cell Signaling Technology	1:1000
AMPKα	2532	Cell Signaling Technology	1:1000
PPARα	AF5301	Affinity Biosciences	1:1000
PPARγ	2443	Cell Signaling Technology	1:1000
C/EBPα	8178	Cell Signaling Technology	1:1000
PGC-1α	A20995	ABclonal Technology	1:1000
SREBP-1	95879	Cell Signaling Technology	1:1000
GAPDH	32233	Santacruz Biotechnology	1:1000

## Data Availability

Data will be made available on request.

## Supplementary Materials

The following supporting information can be downloaded at: https://www.mdpi.com/article/10.3390/molecules31213806/s1, Figure S1: Expanded protein–protein interaction (PPI) network generated using the 57 common targets shared between GA and obesity-associated genes. Additional functionally associated proteins were incorporated using STRING database interactions, resulting in a network comprising 454 nodes and 2195 edges. Node color intensity corresponds to degree values, highlighting highly connected proteins within the network. This expanded network served as the basis for subsequent topological analysis and extraction of the degree-filtered subnetwork shown in Figure 1E, Figure S2: Original uncropped western blot images corresponding to the immunoblots presented in Figure 5, including p-AMPKα, AMPKα, PPARγ, C/EBPα, SREBP-1, PPARα, PGC-1α, and GAPDH in differentiated 3T3-L1 cells treated with geniposidic acid (GA).
