# Supplementary material for: Geniposidic Acid Reduces Lipid Accumulation and Is Associated with Altered AMPK Phosphorylation in 3T3-L1 Adipocytes
Source: Molecules. 2026 Oct 22;31(21):3806. doi: 10.3390/molecules31213806 (PMC13765013; doi:10.3390/molecules31213806)
Supplement: Supplementary file 1 [file molecules-31-03806-s001.zip › molecules-4628871-supplementary.pdf]

**Supplementary Materials**

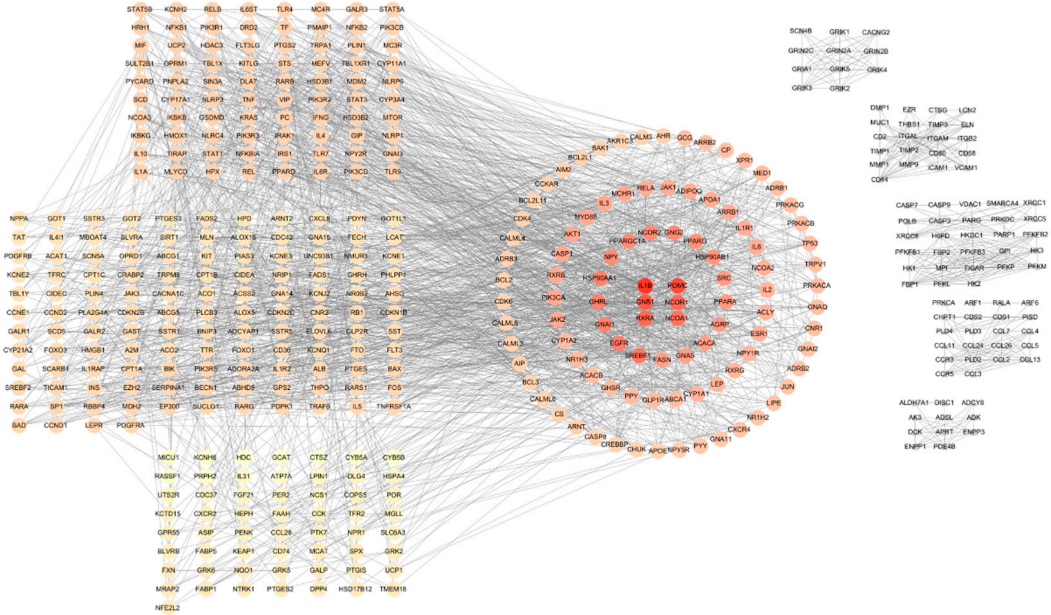

**Figure S1.** Expanded protein–protein interaction (PPI) network generated using the 57 common targets shared between GA and obesity-associated genes.

Additional functionally associated proteins were incorporated using STRING database interactions, resulting in a network comprising 454 nodes and 2,195 edges. Node color intensity corresponds to degree values, highlighting highly connected proteins within the network. This expanded network served as the basis for subsequent topological analysis and extraction of the degree-filtered subnetwork shown in Figure 1E.

3T3L1_Geniposidic Acid_Western whole blot

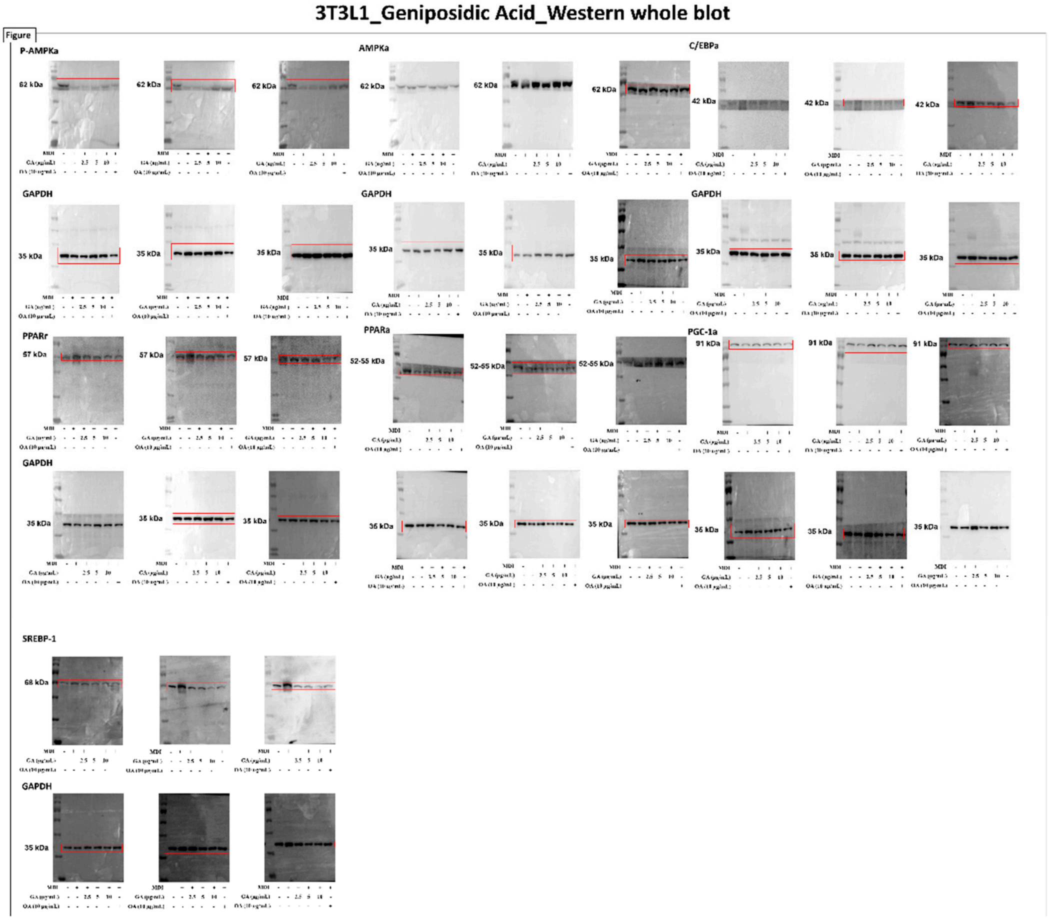


**Figure S2.** Original uncropped western blot images.

Original uncropped western blot images corresponding to the immunoblots presented in Figure 5, including p-AMPKα, AMPKα, PPARγ, C/EBPα, SREBP-1, PPARα, PGC-1α, and GAPDH in differentiated 3T3-L1 cells treated with geniposidic acid (GA)
